# Identification and characterization of a new E3 ubiquitin ligase in white spot syndrome virus involved in virus latency

**DOI:** 10.1186/1743-422X-5-151

**Published:** 2008-12-17

**Authors:** Fang He, Jimmy Kwang

**Affiliations:** 1 Animal Health Biotechnology, Temasek Life Sciences Laboratory, National University of Singapore, 1 Research Link, Singapore, 117604, Singapore; 2 Department of Microbiology, Faculty of Medicine, National University of Singapore, Block MD4, 5 Science Drive 2, Singapore 117597, Singapore

## Abstract

White spot syndrome virus (WSSV) is one major pathogen in shrimp aquaculture. WSSV ORF403 is predicted to encode a protein of 641 amino acids, which contains a C3H2C2 RING structure. In the presence of an E2 conjugating enzyme from shrimp, WSSV403 can ubiquitinate itself *in vitro*, indicating it can function as a viral E3 ligase. Besides, WSSV403 E3 ligase can be activated by a series of E2 variants. Based on RT-PCR and Real time PCR, we detected transcription of WSSV403 in the commercial specific-pathogen-free (SPF) shrimp, suggesting its role as a latency-associated gene. Identified in yeast two-hybrid screening and verified by pull-down assays, WSSV403 is able to bind to a shrimp protein phosphatase (PPs), which was characterized before as an interaction partner for another latent protein WSSV427. Our studies suggest that WSSV403 is a regulator of latency state of WSSV by virtue of its E3 ligase function.

## Background

White spot syndrome virus (WSSV) is a virulent shrimp pathogen responsible for high mortality in cultured shrimp, raising major concerns in the aquaculture industry. Disease outbreaks can reach a cumulative mortality of up to 100% within 3 to 7 days of infection [1]. Its circular dsDNA genome consists of 300 kbp that contains approximately 185 open reading frames (ORFs) [2,3], which is one of the largest viral genomes. Database searches reveal that more than 95% of these ORFs do not have any counterparts in other species and WSSV has thus been placed in a new virus family, the *Nimaviridiae*, genus *Whispovirus *[3].

In the past several years, studies of WSSV mainly focused on the viral structural proteins and more than 30 proteins matching WSSV ORFs have been identified as envelop proteins and collagen-like protein [4-6]. Only a few non-structural genes have been characterized. Three latency-associated genes (LAG) were identified from specific-pathogen-free shrimp by microarray [7]. Among them, ORF89 was found to be a transcription repressor [8] and WSSV427 can interact with a shrimp phosphatase [9]. Microarray has also been employed in WSSV studies to find out three immediate early (IE) genes [10]. At the molecular level, there is little understanding of how WSSV establishes latent infections or of the genes responsible for the transition between latent and lytic infection, which eventually leads to mortality.

Besides, four proteins of WSSV, namely WSSV199, WSSV222, WSSV249 and WSSV403 contain RING-H2 domains [2,11]. A previous study has revealed the involvement of the RING finger domain in specific ubiquitination events by acting as the E3 ubiquitin protein ligase. RING finger domains are subdivided into two subgroups, the C3HC4 (RING-HC) subgroup and the C3H2C3 (RING-H2) subgroup. Among these RING proteins from WSSV, WSSV222 mediates the degradation on a shrimp tumor suppressor as a viral E3 ligase [12] and WSSV249, also acting as an E3 ligase, sequesters the shrimp E2 ubiquitin-conjugating enzyme [11]. To fully display function of RING proteins in WSSV, here we focus on WSSV403, another viral E3 candidate, which is potentially involved in the regulation of WSSV latency.

Specific-pathogen-free (SPF) shrimp are thought to lack WSSV before the three latency-associated genes were identified [7]. Commercialized SPF shrimp (BIOTEC, Bangkok, Thailand) have been tested to be WSSV negative using an IQ2000 WSSV detection kit (Farming IntelliGene Technology Corporation). These shrimp have been grown for 6 generations in a controlled environment without any disease outbreak. Therefore, these SPF shrimp could be used as better research material for WSSV latency study without WSSV contamination compared with normal asymptomatic shrimp, especially in those highly-sensitive methods, such as Real time PCR, which could be used to differentiate latency-assoicated genes from normal genes [7]. Meanwhile, visible symptoms will take place in normal shrimp due to environmental stress rather than virus contamination, raising the possibility that these shrimp contain WSSV in a dormant state [13-15]. In this study, both of normal shrimp and these SPF shrimp were used to study WSSV403 latency associated function.

## Materials and methods

### Reverse transcription PCR and real time PCR

Healthy adult *P. vannamei *weighing around 15 g was verified to be free of WSSV by RT-PCR with primers for VP28 prior to infection. Total RNA from head tissue of four healthy and four infected shrimp was extracted using Trizol reagent (Invitrogen) according to the manufacturer's protocol. After treatment with DNase I the RNA samples were stored in aliquots at -80°C until further use. Subsequently, RT-PCR amplification of WSSV403 was performed with reverse transcriptase (Stratagene) according to the manufacturer's protocol as described before [7]. β-actin specific primers were used as a normalization control for RNA quality and amplification efficiency. Real time PCR was performed using the RNA Master SYBR Green I system and LightCycler (Roche) as recommended by the supplier.

### Expression, purification of proteins and antibody preparation

WSSV403 and 403RING were ligated to pQE30 (Qiagen) using *Bam*HI and *Sal*I sites for construction of expression plasmids. PPs was cloned into pGEX-4T3 vector. 403-transformed *E. coli *M15 (pREP4) cells were cultured in LB with ampicillin (200 μg/ml) at 16°C and induced with 1 mM isopropyl-1-thio-β-D-galactopyranoside (IPTG), while the one of PPs (protein phosphatase) was cultured at 37°C. Bacteria were harvested by centrifugation, resuspended in lysis buffer (New England Biolabs) and lysed by sonication. The expressed proteins were then bound to Ni-NTA beads (New England Biolabs) or GST beads. The purified protein-conjugated beads were then denatured in Laemmli sample buffer prior to SDS-PAGE on a 12% gel and subjected to Western blot.

Guinea pigs were boosted three times with the same quantities of antigen emulsion for WSSV403 every other day for 14 days. Ten days after the final booster injection, the animals were sacrificed by exsanguination and sera were collected.

### Pull-down assays

Cell lysate from WSSV403-expressing *E. coli *was incubated with purified GST-PPs or GST protein (negative control) at 4°C for 2 h. The mixtures were clarified by centrifugation at 1500 × *g *for 10 min, supernatants incubated with fresh GST beads, and then washed in ice-cold wash buffer (100 mM Tris-HCl [pH 8.0], 150 mM NaCl, 5% glycerol, 0.1% Nonidet P-40, 5 mM β-mercaptoethanol) five times at 4°C. The beads were then denatured in Laemmli sample buffer prior to SDS-PAGE and immunoblotting with anti-His_6 _and anti-GST antibodies respectively.

### Ubiquitination assays *in vitro*

E1 and E2 enzymes used in this experiment were purchased from Boston Biochem. *In vitro *ubiquitin conjugation assays were performed in a buffer containing 50 mM Tris-HCl (pH 7.5), 5 mM MgCl and 2 mM ATP. The concentration of protein and enzymes used were as follows: 50 nM E1, 250 nM E2, 5 μg ubiquitin (Sigma), approximately 200 ng of E3 ligase. After 4 h incubation at 30°C the reactions were quenched with Laemmli sample buffer and subjected to electrophoresis on a 10% SDS-polyacrylamide gel. Proteins were transferred to a nitrocellulose membrane for immunoblotting and then probed sequentially using anti-ubiquitin monoclonal P4D1 (1:1000; Santa Cruz Biotechnology) and horseradish peroxidase conjugated rabbit anti-mouse immunoglobulin G (IgG; 1:1000) for *in vitro *assays. Bound antibody was detected using enhanced chemiluminescence reagent (Pierce) and exposure on film.

### Yeast two-hybrid assays

Two-hybrid assays were performed using the Matchmaker GAL4 kit (Clontech). Growth conditions, media, and transformation protocols were as described by the manufacturer. The bait construct pGBKT7-403 and the shrimp cDNA library in pGADT7 were used to cotransform yeast strain AH109. Transformants were selected for growth on -His/-Leu/-Trp dropout medium. The selected colonies were then transferred to -Ade/-His/-Leu/-Trp plates containing 250 μl X-α-gal (2 mg/ml in DMF, Genomax) per 15 cm plate. Blue colonies were selected and cultured in -Ade/-His/-Leu/-Trp broth and lysed with glass beads (Sigma) for plasmid isolation in lysis buffer (2% Triton X-100, 1% SDS, 100 mM NaCl, 10 mM Tris-HCl, pH 8.0, 1 mM EDTA). Isolated plasmids were amplified in *E. coli *DH5α and the target insertions verified by sequencing. Target and bait plasmids were then cotransformed into AH109 to reconfirm the interactions.

## Results

### WSSV403 is a RING-H2 E3 ligase

**Figure 1:**
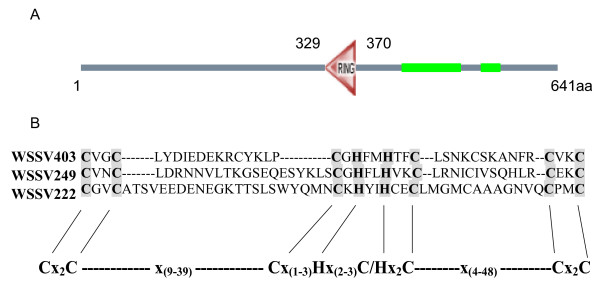
WSSV403 contains a RING domain. (A) Schematic representation of WSSV403 protein by SMART program. RING domain is from 329 aa to 370 aa. Green bars indicated coiled coil regions on WSSV 403. (B) Alignment of the RING portion WSSV403 with other RING proteins identified in WSSV. The WSSV403 RING domain is of the C3H2C3 type.

**Figure 2:**
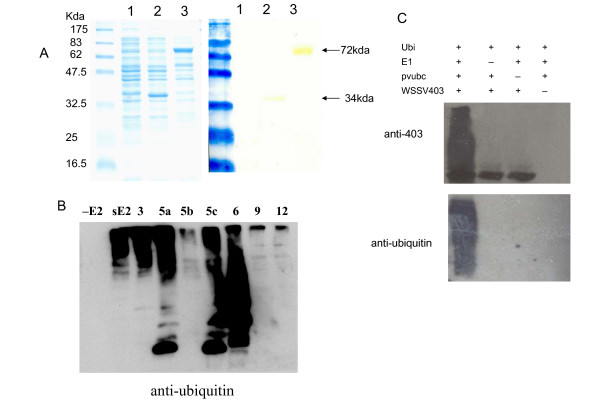
WSSV403 is a viral E3 ubiquitin ligase. (A) Both full-length WSSV403 and 403RING can be expressed in *E. coli *with pQE vector and expression is confirmed by Western blot with anti-histidine antibody. 1: Total cell lysate from un-induced *E. coli*; 2: Total cell lysate from *E. coli *expressing 403RING; 3: Total cell lysate from *E. coli *expressing WSSV403. (B) A panel of different E2 enzymes was screened for activity in the presence of 403RING. The negative control reaction was performed in the absence of E2. -E2: negative control without E2 conjugating enzyme; sE2: shrimp E2 Pvubc; 5a, 5b, 5c, 6, 9 and 12: Ubc 5a, Ubc5b, Ubc5c, Ubc6, Ubc 9 and Ubc 12 are variant E2 enzymes from human. (C) *In vitro *conjugation assay using anti-WSSV403 and anti-ubiquitin antibody P4D1. WSSV403 can be polyubiquitinated in the presence of Pvubc, a shrimp E2 ubiquitin conjugation enzyme.

The full-length *WSSV403 *was cloned from WSSV DNA, encoding a protein of 641 aa. An initial characterization of the putative protein encoded by WSSV ORF403 (AF332093) revealed the presence of a RING finger domain similar to those from WSSV222 and WSSV249 (Fig. 1). The presence of a C3H2C3-type RING finger suggested that WSSV403 belongs to the RING-H2 subgroup and could be involved in ubiquitination. To focus on this RING domain, a RING-containing fragment named *403RING *was cloned from *WSSV403*. 403RING protein (211-494aa) was expressed in *E. coli *by pQE vector (Qiagen) as well as full-length WSSV403 (Fig. 2A). The His_6_-tagged proteins were detected by Western Blot with anti- His_6 _antibody (Qiagen) and purified for *in vitro *ubiquitination assays as previously described [16]. To determine if WSSV403 possessed ubiquitination activity *in vitro *and which, if any, E2 enzyme stimulated this activity, purified 403RING was incubated with a range of different E2 enzymes, including a shrimp E2 Pvubc [16], in the presence of E1, ubiquitin and ATP. Among these E2s we used, 403RING can be strongly activated as an E3 ligase by Pvubc, ubcH3, ubcH5a, ubcH5c and ubcH6 (Fig. 2B), indicating that 403RING can support E3 ligase activity and display a low degree of E2 specificity. Besides 403RING, with Pvubc, the full-length WSSV403 can also be polyubiquitinated by itself (Fig. 2C), confirming its viral E3 function.

### *WSSV403 *is a latency-associated gene

**Figure 3:**
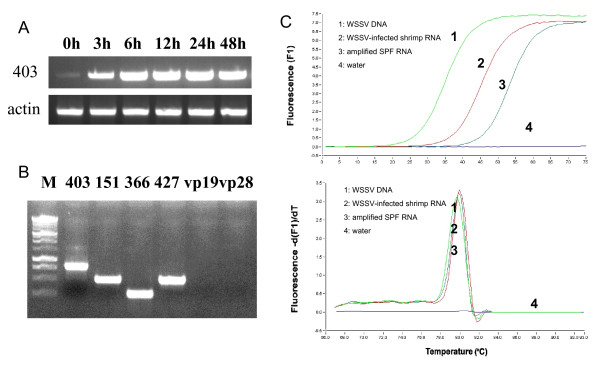
Detection of *WSSV403 *transcript in shrimp. (A) *WSSV403 *transcription was detected in shrimp during WSSV infection by time course RT-PCR. *WSSV403 *transcription was detected in shrimp before WSSV inoculation. (B) *WSSV403 *transcript was found in SPF shrimp RNA by nested RT-PCR. Three latency-associated-genes, *WSSV151*, *366 *and *427*, were indicated as positive controls, while *VP19 *and *VP28 *were used as negative controls. (C) Amplification profiles and dissociation curves of *WSSV403 *in real time PCR using WSSV DNA, total RNA from WSSV-infected shrimp and amplified RNA from SPF shrimp. Water was used as a negative control.

To further study this viral E3 ligase with WSSV, a time course RT-PCR was performed with WSSV-infected shrimp RNA for *WSSV403 *using methods described before [16]. *WSSV403 *transcription was detected in all of WSSV-inoculated samples. And mRNA expression of *WSSV403 *gradually increases after WSSV inoculation. Surprisingly, from normal shrimp RNA sample without WSSV inoculation, *WSSV403 *transcript was also found (Fig. 3A). This result suggests the potential role of *WSSV403 *in latency. Moreover, SPF shrimp samples were tested in RT-PCR for *WSSV403 *expression. With the three identified latency-associated genes, *WSSV151*, *WSSV366 *and *WSSV427 *[7], as positive controls, two-step PCR was employed to amplify *WSSV403 *gene from SPF shrimp cDNA (Fig. 3B). Primers for full-length *WSSV403 *were used in the first step of the PCR and nested PCR was then performed using primers for *403RING*. The amplicon for *WSSV403 *was purified and sequenced for confirmation. Meanwhile, two other genes, *VP19 *and *VP28*, were used as negative controls in this two-step PCR. To further verify this result, SYBR Green real-time RT-PCR was done to identify *WSSV403 *in SPF shrimp. Here, a specific primer for *WSSV403 *was used in reverse transcription of the SPF shrimp RNAs. cDNA from this reverse transcription was further used as the template for Real time PCR. WSSV DNA and WSSV-infected shrimp cDNA were included to this experiment as positive controls. *WSSV403 *was amplified by this approach (Fig. 3C), indicating that *WSSV403 *is a latency-associated transcript for WSSV in SPF shrimp.

### WSSV403 interacts with shrimp phosphatase

**Figure 4:**
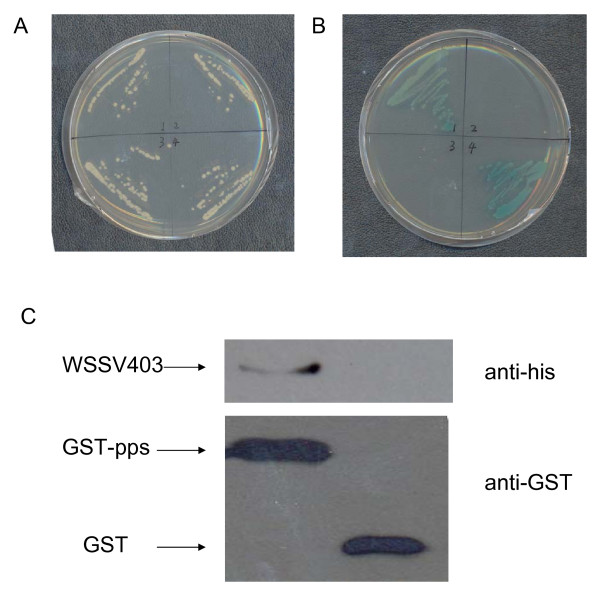
WSSV403 can interact with a shrimp protein phosphatase. WSSV403 was found to interact with shrimp PPs in yeast two hybrid. Cotransformed yeast was screened on -Leu-Trp SD plates (A) and -Leu-Trp-His-Ade plates with x-α-gal (B). 1, yeast cotransformed with pGBK-403 and pGAD-PPs; 2, yeast cotransformed with pGBK-403 and pGAD; 3, yeast cotransformed with pGBK and pGAD-PPs; 4, yeast cotransformed with positive plasmids from the kit (Clontech). (C) Pull-down assays with WSSV403 and GST-PPs. Soluble protein complexes were bound to GST beads and washed under high stringency condition before SDS-PAGE and immunoblot detection of His_6_-WSSV403. Immunoblots were performed with anti-His_6 _or anti-GST.

To understand better this new latent gene of WSSV, yeast two-hybrid was performed with WSSV403 as a bait to screen shrimp cDNA library according to procedures described previously [12]. Among 15 clones we obtained on high-stringent plates, by DNA sequencing, 4 clones were found to encode a protein phosphatase which was identified in our laboratory before [9]. Plasmids extracted from these yeast clones were retransformed to yeast for verification. Blue colonies appeared on high-stringent plates with x-α-gal (Fig. 4A, B), indicating WSSV403 can interact with shrimp protein phosphatase in yeast. To further investigate this phenomenon *in vitro*, pull-down assays were performed by proteins expressed in *E. coli*. Total cell lysates from *E. coli *expressing GST-PPs or His6-WSSV403 was mixed and incubated at room temperature for 2 h. The reaction mixture was clarified by spinning and the supernatant was collected and incubated with GST beads. Protein complex eluted from GST beads was tested by Western blot with anti-his6 antibody. Figure 4C showed that WSSV403 tagged with His6 was detected in samples from GST-PPs, while it was absent in the control test of GST only, indicating WSSV403 can specifically interact with PPs. This result further confirms the physical interaction between WSSV403 and shrimp protein phosphatase. Interestingly, the same shrimp PPs can interact with WSSV427, another latency-associated protein in WSSV, implying that all of the three proteins are involved in WSSV latency regulation pathway.

## Discussion

Viral latency, which is defined operationally as the persistence of the viral genome without production of infectious virions, but with the potential to be activated under certain stimuli, happens in several DNA viruses, such as human cytomegalovirus [17] and Epstein-Barr virus [18]. Here, one novel latency-associated transcript was identified from SPF shrimp during our studies on RING-containing proteins from WSSV. And its viral gene expression was detected in normal shrimp tissue. Taken together with the other three latency-associated-genes of WSSV found previously [7], this report further verifies that viral gene transcription takes place in asymptomatic shrimp, and suggests that WSSV genome is present in SPF shrimp and WSSV latent infection takes place in its host tissue. Though some latency-associated genes can inhibit virus lytic stage to maintain virus latency, such as latent gene vFLIP from Kaposi's Sarcoma-Associated Herpesvirus [19], some other ones contribute to the transit between the latent and lytic stage. For example, the latency-associated transcript gene of herpes simplex virus type 1 (HSV-1) is required for efficient *in vivo *spontaneous reactivation of HSV-1 from latency based on its anti-apoptosis function [20,21]. In our studies, WSSV403 transcription takes place in normal shrimp during the potential latency of WSSV and increases once the lytic stage starts. This finding suggests that WSSV403 expression should contribute to the activation of lytic stage. And such function of WSSV403 could be repressed by certain factors during the virus latent stage, one of which is probably the protein phosphorylation.

The interaction between WSSV403 and shrimp protein phosphatase makes it possible for WSSV403 to be a regulator of latent and lytic infection of WSSV, since the regulation on such kind of proteins by protein phosphatases has been implicated in the latent-lytic life cycle for some other model viruses. In herpes simplex virus, inhibition of protein phosphatase 2B results in a increase in the amount of the regulatory protein ICP0, which leads to efficient virus replication [22]. The switch from latency to viral replication of Epstein-Barr virus is mediated by Zta, the protein product of EBV gene BZLF1. And transcriptional activation of the BZLF1 promoter is greatly augmented by the Ca2+/calmodulin-dependent phosphatase calcineurin [23]. Here, for the interaction between WSSV403 and shrimp PPs, one possibility is that the WSSV403 function depends on its phosphorylation status regulated by the shrimp PPs. WSSV403 E3 function could be activated by dephosphorylation with shrimp PPs. This could lead to WSSV403-mediated ubiquitination on other host proteins in downstream in order to trigger virus replication.

Further, ubiquitination plays important role in viral latency regulation. For example, RING protein ICP0, a regulator of herpes simplex virus during lytic and latent infection, is well-characterized as an E3 ligase [24]. Latent membrane protein 2A of Epstein-Barr virus utilizes ubiquitin-dependent processes to modulate cellular signaling pathways involved in latency regulation [25]. As a RING-containing E3 ubiquitin ligase, WSSV403 is able to interact with its substrates besides E2 conjugating enzymes and to mediate degradation of the substrate, which enables it to regulate other proteins in downstream via ubiquitination pathway. Thus, another model for WSSV403 involved in WSSV latency regulation could be that shrimp PPs is the potential substrate for WSSV403 in ubiquitination. WSSV403 could down-regulate shrimp PPs via ubiquitin-mediated degradation. This reaction could inhibit PPs-mediated dephosphorylation on other viral or host proteins in down-stream, which could be WSSV427. In WSSV, latency-associated protein WSSV427 is another interaction partner for the shrimp PPs [9], indicating the systematic regulation between viral latent proteins and host proteins. The detailed relation of WSSV403, the shrimp protein phosphatase and WSSV427 will be further explored in future studies. Here, this study identified RING protein WSSV403 as a candidate of latency regulator, which paves the way for clarifying the mechanism of transit from latency to lytic stage in WSSV.

## Conclusion

Results here indicate that WSSV403 is a new viral E3 ubiquitin ligase in WSSV. Its latent gene transcription and PPs binding activity suggest that WSSV403 is a regulator of latency state of WSSV by virtue of its E3 ligase function.

## Competing interests

The authors declare that they have no competing interests.

## Authors' contributions

FH carried out the experiments, analyzed the data and drafted the manuscript and JK contributed to the experimental design of the study and critical analysis of the data.
